# The second to fourth digit ratio (2D:4D): a risk factor of migraine and Tension-type headache

**DOI:** 10.1186/s10194-015-0494-8

**Published:** 2015-02-11

**Authors:** Wei Xie, Mianwang He, Ruozhuo Liu, Zhao Dong, Jingdan Xie, Dan Wang, Shengyuan Yu

**Affiliations:** Department of Neurology, International Headache Center, Chinese PLA General Hospital, Fuxing Road 28, Haidian District, Beijing, 100853 China

**Keywords:** Migraine, Tension-type headache, Digit ratio, 2D:4D, Sex steroids

## Abstract

**Background:**

Migraine and Tension-type headache (TTH) are common and disabling primary headache disorders. They are more prevalent in females. The second to fourth digit ratio (2D:4D) is sexually dimorphic in humans and is considered to be a marker for the balance of prenatal testosterone and estrogen exposure. Therefore, we investigated the hypothesis that prenatal sex steroids constitute an independent risk factor for adult headaches later in life.

**Methods:**

A total of 891 individuals (303 males, 588 females) of 18–68 years in age (a mean of 37.0 ± 10.1 years), including 279 migraine, 300 TTH, and 312 healthy subjects were enrolled. The 2D:4D ratio was measured by a single investigator using a digital Vernier caliper.

**Results:**

The females in the migraine group had lower 2D:4D ratios than those in the control group (left hand: 0.947 ± 0.034 vs. 0.955 ± 0.038, p = 0.048; right hand: 0.952 ± 0.035 vs. 0.965 ± 0.037, p = 0.001). There was a similar relationship between the TTH group and the control group (left hand: 0.946 ± 0.034 vs. 0.955 ± 0.038, p = 0.034; right hand: 0.954 ± 0.035 vs. 0.965 ± 0.037, p = 0.005), although this was not observed in males. Males showed lower 2D:4D ratios than females for the right hand in the control group (0.954 ± 0.039 vs. 0.965 ± 0.037, p = 0.015). No significant difference was found in the left hand.

**Conclusions:**

These results suggested that the 2D:4D ratio is a risk factor of migraine and TTH and that the balance of prenatal estrogen and testosterone in utero may impact adult primary headache disorders.

## Background

Migraine is among the most common and disabling primary headache disorders that recur with a series of complex symptoms. It is a major cause of the disturbance of the brain system in behavioral and/or physiologic aspects [1]. Globally, the prevalence of the adult population with an active headache disorder is 46% for headaches in general and 11% for migraines. A migraine is two to three times more prevalent in women than in men [2]. In China, an epidemiology survey showed that the one-year prevalence of migraine was 9.3% (males: 5.9%; females: 12.8%) [3].

Considerable scientific evidence indicated that sex steroids have complex, dynamic effects in widespread cerebral and brainstem regions that have been implicated in the pathophysiology of migraine. For example, Andrew G et al. found that several neurotransmitter systems that were implicated in migraine varied with reproductive steroid levels during the reproductive cycle and that estrogen stabilization may provide effective treatment in susceptible women [4]. Pfaff D et al. reported that estradiol receptors are abundant in the dorsal raphe in neurons adjacent to serotoninergic cells and that serotonin may play a role in both the development and relief of migraine [5]. Other sex steroids also may have the potential to regulate migraine. Eikermann-Haerter K et al., using a mouse model of familial hemiplegic migraine (FHM), found that testosterone suppresses cortical spreading depression (CSD), which is considered to be one of the important pathophysiological mechanisms in the development of migraine [6].

In addition to migraine, TTH shows significant sex-related differences in incidence and prevalence [3,7]. Sex steroids also may have been implicated in the pathophysiology of TTH.

The 2D:4D ratio is sexually dimorphic in humans [8]. It is determined in a relatively narrow time window toward the end of the first trimester of fetal development and changes little with subsequent growth [9,10]. The 2D:4D ratio is considered to be a negative correlate of prenatal testosterone and a positive correlate of prenatal estrogen [11]. The digit ratio varies by ethnicity and country [12]. A broad range of research links the 2D:4D ratio and later behavioral and physiological outcomes together. The latter include sperm counts, autism, personality characteristics, myocardial infarction (MI), coronary artery disease (CAD), myopia, adult lung function and amyotrophic lateral sclerosis (ALS) and so on [13-15]. However, the relationship between the 2D:4D ratio and primary headache disorders is unknown.

Therefore, the aim of the current study was to investigate the relationship between the 2D:4D ratio and migraine and TTH. From above, it is possible to make the assumption that estrogenic forms of 2D:4D (i.e., higher values) will correlate positively with migraine and TTH.

## Methods

### Participant selection

The study was approved by the Chinese Ministry of Health and the Ethics Committee of the Chinese People’s Liberation Army (PLA) General Hospital, Beijing.

Patients, who were diagnosed as migraine or TTH at the International Headache Center of Chinese PLA General Hospital from March to August of 2014, were invited to participate in the study. A total of 286 patients who were suffering from migraine and 306 who were suffering from TTH were enrolled in the study. We selected randomly 318 healthy people from many living and working in Northern and Eastern China came to the medical center of our hospital for routine medical checks as controls and they received the same type of invitation as patients did. The subjects, who were excluded from the study were individuals who might have been of minority ethnicities rather than Han Chinese, had more than one type of primary headache, and had secondary headaches and with finger deformity.

### Diagnostic of primary headache disorders

The International Classification of Headache Disorders, Third Edition (ICHD-3 beta) criteria [16] was applied for diagnosis of primary headache disorders. In order to increase the diagnostic accuracy, at least two headache specialists confirmed the final diagnosis.

### Measurement of digit ratio

A photograph of both hands were taken using a digital camera [13]. All photographs were taken at the same distances from the midpoint of the hand. The lengths of the fingers were measured by a single investigator using a digital Vernier caliper that is accurate to 0.01 mm [17]. Measurements were taken from the bottom crease to the tip of the finger on the palmar surface of hands. The length of the index finger was divided by the length of the ring finger to obtain the 2D:4D ratio.

### Statistical analysis

The data were analyzed using SPSS version 17.0. One-way analysis of variance (ANOVA) was used to compare numerical variables of the three study groups. Pearson x^2^ was used to compare difference of gender and family history in three groups. Paired-Samples T Test was used to compare the 2D:4D ratio between right hand and left hand in total subjects. The Independent-Samples T Test was used to compare 2D:4D between male and female in both hands in control group. The statistical significance was set at P < 0.05.

## Results

### Participant clinical characteristics

Of the 910 participants, 15 people were not Han Chinese (6 people in migraine, 5 people in TTH and 4 people in control group) and 4 people had former hand lesions (1 people in migraine, 1 people in TTH and 2 people in control group) were excluded, leaving 891 subjects included in the present study. The clinical characteristics of all 891 participants (303 males, 588 females) of 18–68 years in age (a mean of 37.0 ± 10.1 years) are summarized in Table 1. All subjects were photographed. The subjects came from 22 regions of China. Most of them lived in Northern and Eastern China, including Beijing (n = 120, 13.5%), Hebei (n = 141, 15.8%), Inner Mongolia (n = 89, 10.0%) and so on. There were no significant differences in age, gender and birth place among the three groups. More patients with migraine and TTH have a family history than those without headache (28.3% vs. 1.9%, 19.3% vs. 1.9% respectively).

**Table 1:** **The study sample’s demographic data and 2D:4D ratios**

**Characteristic**	**Migraine**	**TTH**	**Non**	**p value**
Number	279	300	312	
Gender				0.118
Males	83 (29.7%)	102 (34%)	118 (37.8%)	
Females	196 (70.3%)	198 (66%)	194 (62.2%)	
Age	37.3 ± 9.5	37.0 ± 8.8	36.8 ± 11.6	0.167
Family history	79 (28.3%)	58 (19.3%)	6 (1.9%)	0.000
Total				
Left 2D:4D	0.946 ± 0.034^*†^	0.947 ± 0.035^*†^	0.954 ± 0.040^†^	0.013
Right 2D:4D	0.953 ± 0.035^*^	0.954 ± 0.036^*^	0.961 ± 0.038	0.012
Males				
Left 2D:4D	0.944 ± 0.034	0.950 ± 0.036	0.953 ± 0.043	0.267
Right 2D:4D	0.953 ± 0.034	0.954 ± 0.037	0.954 ± 0.039^‡^	0.990
Females				
Left 2D:4D	0.947 ± 0.034^*^	0.946 ± 0.034^*^	0.955 ± 0.038	0.030
Right 2D:4D	0.952 ± 0.035^*^	0.954 ± 0.035^*^	0.965 ± 0.037	0.001

^*^Significant difference for migraine versus Non、TTH versus Non on both hands in total and female subjects (p < 0.05).

^†^Significant difference for right hand versus left hand in total subjects (p < 0.05).

^‡^Significant difference for male versus female in right hand in control group (p < 0.05).

### The difference in the 2D:4D ratio

The 2D:4D ratio in both hands was lower for females in the migraine group than in the control group (left hand: 0.947 ± 0.034 vs. 0.955 ± 0.038, p = 0.048 and right hand: 0.952 ± 0.035 vs. 0.965 ± 0.037, p = 0.001). There was a similar relationship between the TTH group and the control group (left hand: 0.946 ± 0.034 vs. 0.955 ± 0.038, p = 0.034 and right hand: 0.954 ± 0.035 vs. 0.965 ± 0.037, p = 0.005). However, this was not observed in males. There was no difference in the 2D:4D ratio between migraine and TTH in total (left hand: 0.946 ± 0.034 vs. 0.947 ± 0.035, p > 0.05 and right hand: 0.953 ± 0.035 vs. 0.954 ± 0.036, p > 0.05). Males had a lower mean digit ratio than females for the right hand in the control group (0.954 ± 0.039 vs. 0.965 ± 0.037, p = 0.015), but this was not observed for the left hand. There was a significant difference in the 2D:4D ratio between the right and the left hand in the total of all subjects (left vs. right was 0.949 ± 0.037 vs. 0.956 ± 0.037, p = 0.000) (Table 1).

## Discussion

The digit ratio (2D:4D) is sexually dimorphic. It is determined as early as the 14th week of fetal life and remains unchanged at puberty [18]. Hox genes play a key role between fetal sex hormone and digit formation, which is essential for the differentiation of both the urinogenital system and the digit [19]. Manning et al. reported that females had higher 2D:4D ratios than males (right and left hands) after measuring both hands of 153 men and 153 women in the North and North-West of England [20]. Rothkopf’s study in America obtained the same result [21]. In our study, the male 2D:4D ratio was smaller than the female 2D:4D ratio for the right hand in healthy controls, although this was not observed for the left hand. This result is consistent with the previous studies by Wu, who found significant difference in the digit ratio between men and women only for the right hand in the control group (P < 0.001) [22]. However, Yang et al. found that men had a significantly lower 2D:4D ratio than women in China for both hands (p < 0.0001) [23]. Part of the difference in values may be due to methodological differences in the surveys. Yang’s studies used direct measurement for digit ratio, whereas Wu’s study and the present study used digital photographs for measurement. Studies have shown that the mean 2D:4D ratio measured from photocopies tends to be lower than that from direct finger measurement and the finger length differences between two measurement techniques could result from transforming three-dimensional fingers into two-dimensional photocopy images [24]. A large population-based study is required to determine the digit ratio of Han ethnicity for purposes of comparison.

The present study showed that the 2D:4D ratio negatively correlated to migraine and TTH in women. The lower digit ratio, which is indicative of exposure to higher prenatal testosterone and lower prenatal estrogen, was associated with Migraine and TTH in women [11].

However, this association was in the direction opposite to what had been expected (i.e., higher, more “feminine” ratios were associated with a prevalence of migraine and TTH). It is difficult to interpret this phenomenon, but low estrogen levels implicated in the pathophysiology process of migraine appear to be increasingly corroborated by clinical findings. For example, Sances G et al. reported that estrogen levels were elevated in early pregnancy, but dropped sharply in postpartum. Migraine was seen to improve during pregnancy. 80 percent of 47 migraineur without aura (MO) experienced complete remission. After childbirth, migraine recurred in almost all of the women sufferers [25]. In another study, Calhoun AH et al. used an oral contraceptive containing 20 micrograms of ethinyl estradiol on days 1 to 21, supplemented with 0.9 mg conjugated equine estrogens on days 22 to 28 to treat 11 women with menstrual-associated migraine. They found that almost all patients achieved at least a 50% reduction in the number of headache days per cycle and the weighted headache score [26]. These findings seem to explain this phenomenon. Our findings in this regard dovetail with the hypothesis that suggests that high prenatal testosterone levels are implicated in the etiology of migraine put forward by Geschwind and Galaburda who, however, did not analyze the cause [27].

Not only physiological factors, but also psychological factors, might play important roles in the mechanism of migraine and TTH [7]. Patients with migraine often endorse higher levels of neuroticism, or susceptibility to experience negative effects, than non-migraineurs [28]. Cao et al. concluded that patients with migraine without aura, as well as TTH patients, obtain higher neuroticism scores than do controls [29]. We suspect that there may be some relationship between the personality trait of neuroticism and migraine and TTH. At the same time, Herrmann WM et al. found that estrogen can lead to a decrease in neuroticism according to the Eysenck Personality Model [30]. If high digit ratios are related to higher estrogen, and lower estrogen could be considered to be a risk factor for neuroticism, then estrogen, digit ratio, neuroticism and headache might be related. We suspect that Migraine and TTH attacks in adult life might be partly prenatally programmed by long-lasting effects of sex hormones on the personality trait of neuroticism. If so, it may explain the difference of digit ratio between two headache groups and control group and why no difference was found between migraine and TTH.

Future research is likely to discover the links between the 2D:4D ratio and personality.

One of the most significant limitations of our study was the inability to take all other sex hormones into the scope of analysis. The relationship among different sex hormones (i.e., estrogens, testosterone, progesterone, follicle-stimulating hormone (FSH), luteinizing hormone is complex and should be considered as a whole in order to properly evaluate the role of sex hormones in headache.

## Conclusion

Our study demonstrated that women with lower digit ratios (2D:4D) for both hands tend to suffer migraine and TTH. There is no significant difference between male migraine and TTH suffers and controls in the 2D:4D ratio. The results suggest that digit ratio is a risk factor of migraine and TTH and that the balance of prenatal estrogen and testosterone in utero may impact adult primary headache disorders with physiological and psychological factors. Further research including a wider range of sex hormones will improve the understanding of mechanisms that underlie sex-related differences in headache prevalence.
